# Value of CT features for predicting EGFR mutations and ALK positivity in patients with lung adenocarcinoma

**DOI:** 10.1038/s41598-021-83646-7

**Published:** 2021-03-11

**Authors:** Xiaoyu Han, Jun Fan, Yumin Li, Yukun Cao, Jin Gu, Xi Jia, Yuhui Wang, Heshui Shi

**Affiliations:** 1 Department of Radiology, Union Hospital, Tongji Medical College, Huazhong University of Science and Technology, 1277 Jiefang Rd, Wuhan, Hubei Province 430022 The People’s Republic of China; 2 Hubei Province Key Laboratory of Molecular Imaging, Wuhan, 430022 China; 3 Department of Pathology, Union Hospital, Tongji Medical College, Huazhong University of Science and Technology, 1277 Jiefang Rd, Wuhan, Hubei Province 430022 The People’s Republic of China

**Keywords:** Lung cancer, Cancer, Oncology

## Abstract

The aim of this study was to identify the relationships of epidermal growth factor receptor (EGFR) mutations and anaplastic large-cell lymphoma kinase (ALK) status with CT characteristics in adenocarcinoma using the largest patient cohort to date. In this study, preoperative chest CT findings prior to treatment were retrospectively evaluated in 827 surgically resected lung adenocarcinomas. All patients were tested for EGFR mutations and ALK status. EGFR mutations were found in 489 (59.1%) patients, and ALK positivity was found in 57 (7.0%). By logistic regression, the most significant independent prognostic factors of EGFR effective mutations were female sex, nonsmoker status, GGO air bronchograms and pleural retraction. For EGFR mutation prediction, receiver operating characteristic (ROC) curves yielded areas under the curve (AUCs) of 0.682 and 0.758 for clinical only or combined CT features, respectively, with a significant difference (*p* < 0.001). Furthermore, the exon 21 mutation rate in GGO was significantly higher than the exon 19 mutation rate(*p* = 0.029). The most significant independent prognostic factors of ALK positivity were age, solid-predominant-subtype tumours, mucinous lung adenocarcinoma, solid tumours and no air bronchograms on CT. ROC curve analysis showed that for predicting ALK positivity, the use of clinical variables combined with CT features (AUC = 0.739) was superior to the use of clinical variables alone (AUC = 0.657), with a significant difference (*p* = 0.0082). The use of CT features for patients may allow analyses of tumours and more accurately predict patient populations who will benefit from therapies targeting treatment.

## Introduction

Lung cancer is one of the leading causes of cancer-related death worldwide. In China, approximately 733,300 patients were diagnosed with lung cancer in 2015, with 610,200 deaths, and the number of lung cancer-related deaths is expected to exceed one million by 2025^1,2^. In recent decades, the emergence of new therapies targeting signalling pathways activated by genetic alterations has revolutionized a new treatment approach for non-small-cell lung cancer (NSCLC), especially adenocarcinoma. The two most common druggable targets are epidermal growth factor receptor (EGFR) mutations and anaplastic lymphoma kinase (ALK) rearrangement in lung adenocarcinomas. EGFR mutations are associated with tumour sensitivity to EGFR-tyrosine kinase inhibitors (TKIs)^3^. For tumours with mutations in exons 18, 19, 21, and 20 of the EGFR gene, 80% gefitinib is effective ^4,5^, but it is useless for tumours with EGFR wild-type mutations^6^. Crizotinib was the first drug approved for adenocarcinomas harbouring ALK rearrangement^7^. Randomized clinical trials have demonstrated longer progression-free survival (PFS) following treatment with TKIs than chemotherapy in advanced NSCLC harbouring EGFR mutations or ALK rearrangements^7,8^. Therefore, it is critical to determine EGFR mutation and ALK statuses prior to the use of TKIs in patients with adenocarcinoma. Two types of methods are currently available: “screening” assays and “specific” methods^9,10^. However, both methods for detecting EGFR mutations or ALK status are costly and not feasible for every patient with lung cancer. Accordingly, clinical factors are needed to enrich the analysis of gene status in patients with nonresectable lung adenocarcinomas by computed tomography (CT) because this imaging is readily available (Table 1).

**Table 1:** CT features for lung adenocarcinoma.

Variable	Definition
Type	Central, tumour located in the segmental or more proximal bronchi; peripheral, tumour located in the subsegmental bronchi or more distal airway
Location	The distribution of each lesion in the lung was recorded, including left upper lobe, left lower lobe, right upper lobe, right middle lobe and right lower lobe
Tumour size	Longest diameter of the tumour in MPR images
Texture	Predominantly solid, Tumour solid component/ground glass component > 0.5; Predominantly ground glass opacity, tumour solid diameter/ground glass diameter ≤ 0.5
Ground glass opacity	Ground glass dense nodules with internal vessels and bronchi visible
Mix ground glass opacity	Composition of both ground glass opacity and solid
Pure ground glass opacity	Composition of ground glass opacity only
Shape	Indicated as lobulated, others (round, or oval)
Lobulated	The surface of the tumour showed as multiple arc-shaped projections
Spiculate	Evaluated in the lung window, and indicated as different degrees of spinous or burr-like protrusions at the tumour margin
Margin definition	Evaluated in the lung window, and indicated as well-defined, or poor-defined
Air bronchogram	Tube like or branched air structure within the tumour
Bubble-like lucency	The 1–3 mm of air density area within the mass
Margins	Evaluated in the lung window, and indicated as smooth, or spiculated
Heterogeneity	Evaluated in the soft tissue window, and heterogeneity indicated as the difference of CT values in tumour was greater than 20HU
Pleural attachment	Retraction of the pleura towards the tumour
Thickening of the adjacent pleura	Thickening of the adjacent fissural or peripheral pleura pleura
Pleural effusion	Presence or absence of pleural effusion
Cavitation	Presence or absence of cavitation
Intramodular calcifications	Presence or absence of calcifications
Necrosis	Low-density area in the tumour, without enhancement in enhance CT
Peripheral emphysema	Presence or absence of peripheral emphysema
Peripheral fibrosis	Pulmonary fibrosis around the tumour
Vascular convergence	Convergence of vessels to the tumour, applied to the peripheral tumours
Enhancement	“mild” = 0–20 HU; “moderate” = 20–40 HU, “marked” > 40 HU
Lymphadenopathy	Presence or absence of lymphadenopathy thoracic lymph nodes (hilar or mediastinal) with short-axis diameter greater than 1 cm
Distance metastases	Including metastasis in bone, brain, liver metastasis, etc. Detected at the same time as the diagnosis of primary tumour or occurred within 6 months’ following up after surgery

Although several studies have investigated relationships between CT imaging features and EGFR mutation^11–15^ and ALK status^16–19^, such associations in lung adenocarcinoma are conflicting due to the small sample sizes. Glynn et al. and Sugano et al.^11,12^ found no significant association between CT features and EGFR mutations, whereas Liu et al.^13^ reported that a ground glass opacity (GGO) appearance and 15 other CT features were significantly associated with EGFR mutations. Furthermore, the difference in CT features between EGFR exon 21- and 19-mutated adenocarcinomas remains unclear, since it has been proven that TKIs show different targeted effects in cases of EGFR exon 21 and -19 mutations^20^.

Consequently, our study reviewed data for 827 patients with adenocarcinoma to assess the association between CT and clinical characteristics and EGFR and ALK mutations. We also explored the difference in clinical and CT features between EGFR exon 21- and 19-mutated adenocarcinomas.

## Results

### Clinical characteristics

**Figure 1:**
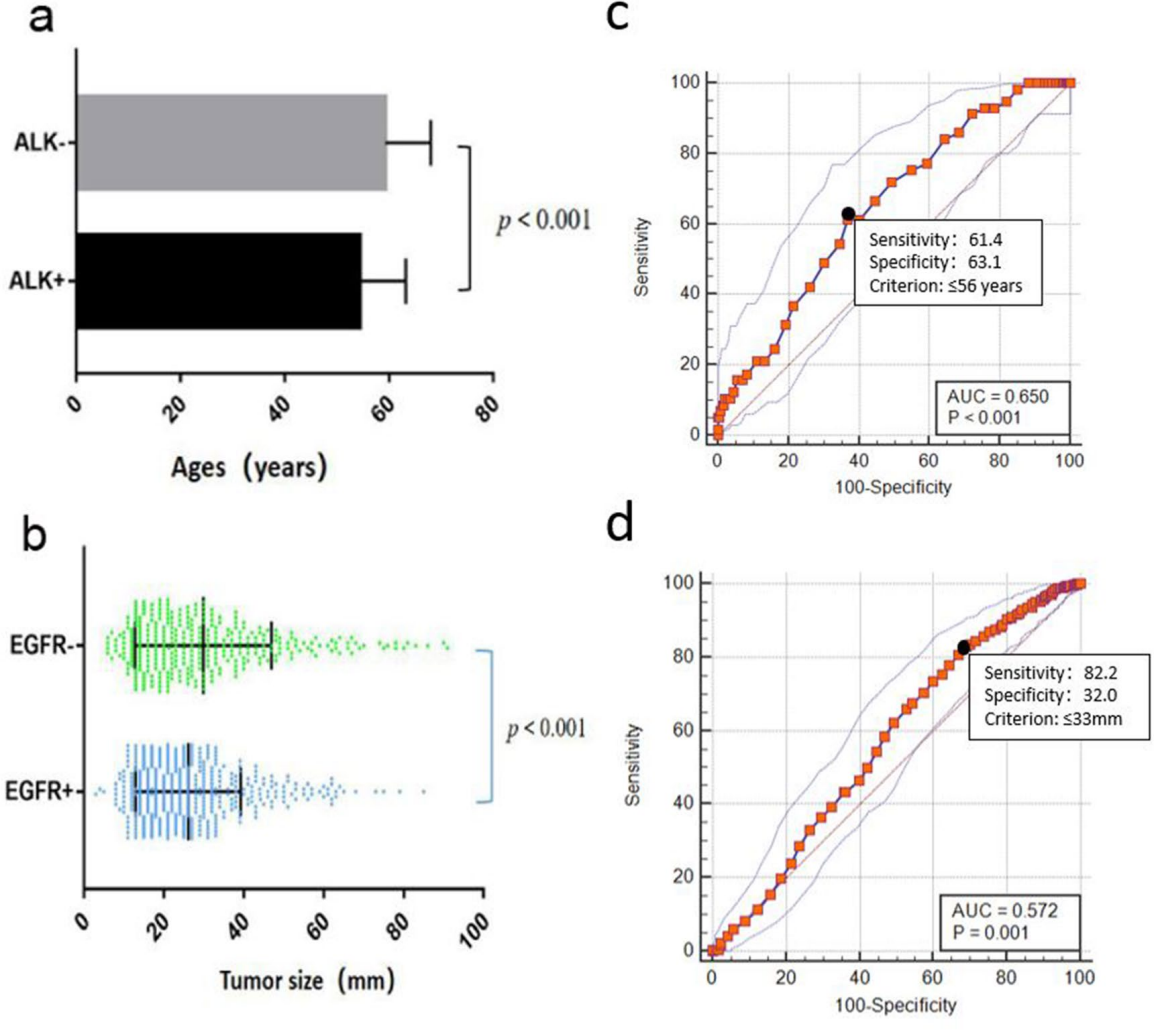
(**a**) Ages of ALK-positive (ALK +) patients compared to ALK-negative (ALK-) patients. Patients; (**b**) tumour size between wild-type EGFR (EGFR-) and mutated EGFR (EGFR +); (**c**) receiver operating characteristic curves of age for predicting ALK positivity; (**d**) receiver operating characteristic curves of the tumour maximum diameter for predicting EGFR mutations.

A total of 827 eligible patients (average age, 59 ± 9 years; 418 males) were included in the study, and their clinical and pathological characteristics are summarized in Table 2. EGFR mutations were found in 489 (59.1%) patients, and exon 21, 19, 20, and 18 mutation rates were 49.5%, 44.0%, 3.1% and 3.4%, respectively. ALK positivity was found in 57 (7.0%) patients. Six patients had concomitant EGFR mutations and ALK positivity (0.7%). EGFR mutations were more common in females than in males (*p* < 0.001) and in those who had never smoked (*p* < 0.001). No significant association was found between age or TNM stage and EGFR mutation (*p* = 0.320 and *p* = 0.831, respectively). Pathologically, EGFR mutation was associated with a high frequency of the lepidic predominant subtypes (*p* < 0.001) and a low frequency of lymph node metastasis proven by surgery (*p* = 0.006). Pleural invasion did not differ between patients with wild-type and mutant disease (*p* = 0.268). ALK positivity was found more frequently in younger patients (*p* < 0.001) (Fig. 1a), and the optimal cut-off value for age was 56 years old (Fig. 1c). A high frequency of solid growth or mucus patterns in ALK-positive tumours was observed in the present study. There was no significant difference in sex, smoking history, TNM stage, lymph node metastasis or pleural invasion between the ALK-positive and ALK-negative groups (Table 2).

**Table 2:** Association between clinical characteristics and EGFR and ALK status in adenocarcinoma.

Variable	EGFR + N (%)	EGFR- N (%)	Total N (%)	*P*	ALK + N (%)	ALK − N (%)	Total N (%)	*P*
Age (years)	59 ± 8	59 ± 9		0.320	54 ± 9	59 ± 9	59 ± 9	< 0.001*
Sex				< 0.001*				0.131
Male	195 (39)	223 (66)	418 (51)		23 (40)	395 (51)	418 (51)	
Female	294 (61)	115 (34)	409 (49)		34 (60)	375 (49)	409 (49)	
Smoking history	109 (23)	165 (49)	274 (33)	< 0.001*	19 (33)	255 (33)	274 (33)	1.000
TNM stage^&^				0.831				0.213
I–II	272 (56)	191 (57)	463 (56)		27 (47)	436 (57)	463 (56)	
III–IV	217 (44)	147 (43)	364 (44)		30 (53)	334 (43)	364 (44)	
Histological subtype								
Lepidic predominant^$^	48 (10)	11 (3)	59 (7)	< 0.001*	4 (7)	55 (7)	59 (7)	1.000
Others subtypes^@^	441 (90)	327 (40)	768 (93)		53 (93)	715 (93)	768 (93)	
Solid or Mucinous	43 (9)	106 (13)	149 (18)	< 0.001*	27 (47)	122 (16)	149 (18)	< 0.001*
Others subtypes^%^	446 (91)	232 (69)	678 (82)		30 (53)	648 (84)	678 (82)	
Acinar predominant	261 (65)	148 (44)	409 (49)		16 (28)	390 (51)	409 (49)	
Papillary predominant	128 (26)	63 (18)	324 (39)		6 (11)	318 (41)	324 (39)	
Solid predominant	36 (7)	83 (25)	119 (14)		17 (30)	102 (13)	119 (14)	
Mucinous predominant	7 (1)	23 (7)	30 (4)		10 (18)	20 (3)	30 (4)	
Micropapillary	8 (2)	7 (2)	15 (2)		3 (5)	12 (2)	15 (2)	
Sieve predominant	1 (0.2)	3 (1)	4 (0.5)		1 (2)	3 (0.4)	4 (0.5)	
Lymph node metastasis	134 (27)	101 (30)	235 (28)	0.006*	18 (32)	217 (28)	235 (28)	0.426
Pleural invasion	142 (29)	86 (25)	228 (28)	0.268	18 (32)	210 (28)	228 (28)	0.539

* *P* values < 0.05 were based on comparisons between the two groups;&According to the IASLC 8th TNM Lung Cancer Staging System; $ Lepidic predominant includes: adenocarcinoma in situ, minimally invasive adenocarcinoma, and lepidic predominant invasive adenocarcinoma;@ Other subtypes include: acinar, papillary, micropapillary, and solid predominant adenocarcinoma, as well as variants of invasive mucinous adenocarcinoma;% other subtypes include: lepidic predominant acinar, papillary, micropapillary, as well as variants of invasive adenocarcinoma; EGFR, epidermal growth factor receptor; ALK, anaplastic large-cell lymphoma kinase EGFR + , EGFR mutation; EGFR-, EGFR wild type mutation; ALK + , ALK positive; ALK-, ALK negative.

### Interobserver agreement of CT interpretations

The intraclass correlation coefficient for maximum tumour diameter was good at 0.963 (95% CI: 0.898, 0.978). The concordance between the two observers was also good, with k coefficients ranging between 0.613 and 0.984 (Table 3).

**Table 3:** Analysis of interreader agreement percent of concordance and kappa of agreement.

CT features	N (% of concordance)	Kappa (95%CI)	Kappa interpretation
Shape	767/827	0.726 (0.655–0.788)	Substantial
Location	803/827	0.828 (0.724–0.906)	Almost perfect
Texture	788/827	0.847 (0.794–0.893)	Almost perfect
Bubblelike lucency	787/827	0.797 (0.727–0.852)	Almost perfect
Margins	777/827	0.851 (0.807–0.888)	Almost perfect
Vascular convergence	758/827	0.767 (0.707–0.818)	Almost perfect
Air bronchogram	762/827	0.827 (0.781–0.867)	Almost perfect
Cavitation	809/827	0.839 (0.761–0.909)	Almost perfect
Pleural retraction	821/827	0.984 (0.969–0.995)	Almost perfect
Pleural effusion	797/827	0.926 (0.893–0.950)	Almost perfect
Spiculate	782/827	0.613 (0.511–0.711)	Substantial
Calcifications	785/827	0.626 (0.510–0.714)	Substantial
Enhancement degree	215/360	0.748 (0.677–0.816)	Substantial
Peripheral emphysema	801/827	0.894 (0.853–0.928)	Almost perfect
Peripheral fibrosis	779/827	0.860 (0.891–0.899)	Almost perfect
Lymphadenopathy	794/827	0.884 (0.842–0.920)	Almost perfect
Thickening pleura	789/827	0.749 (0.673–0.818)	Substantial
Heterogeneity	773/827	0.873 (0.828–0.902)	Almost perfect
Necrosis	747/827	0.694 (0.623–0.751)	Substantial
Metastasis	793/827	0.908 (0.874–0.939)	Almost perfect

### Correlation of EGFR mutations and ALK positivity with CT features

**Figure 2:**
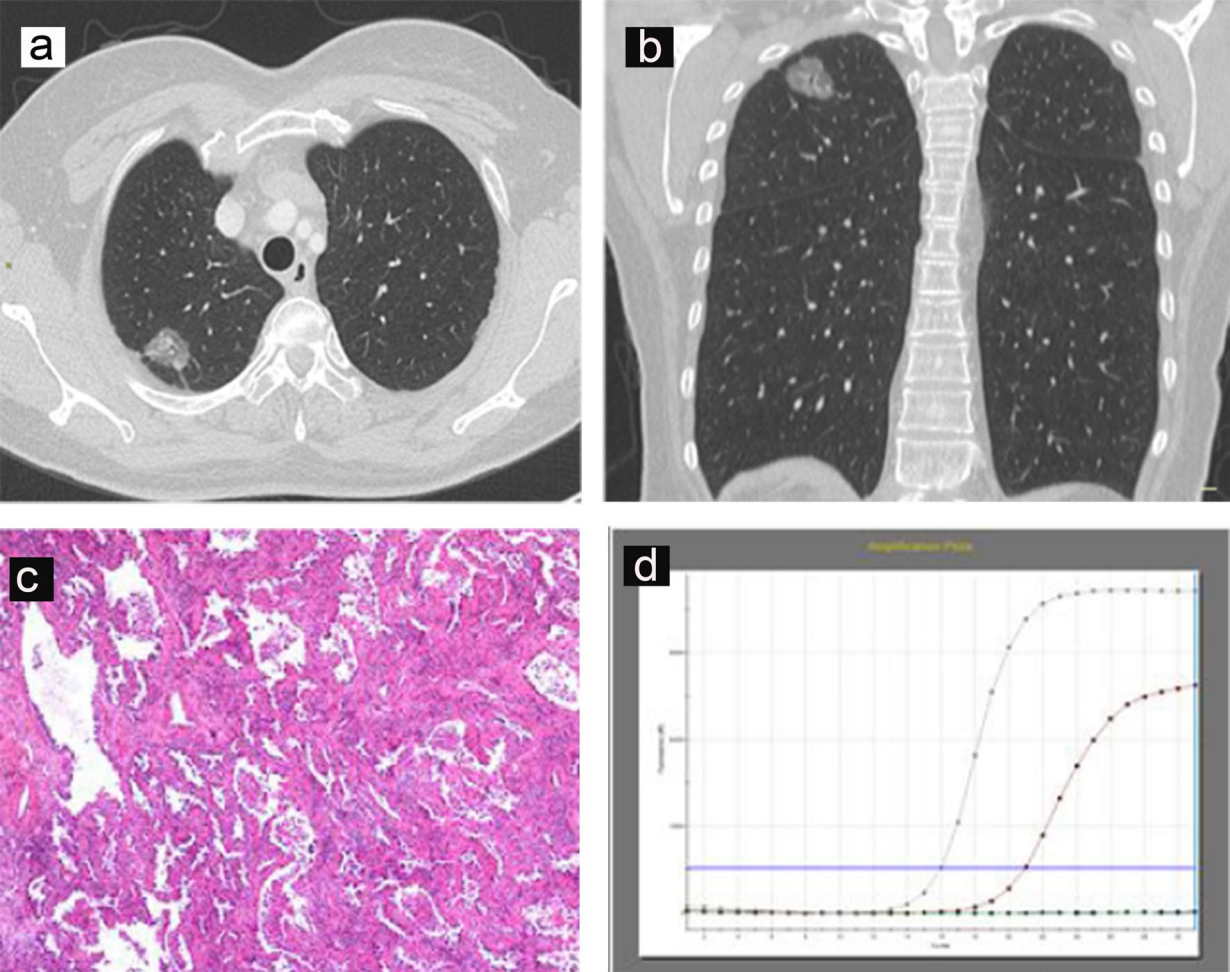
A 53-year-old female with primary lung adenocarcinoma with EGFR mutation and ALK negative in the right upper lobe (**a**,**b**) that showed a mixed ground grass opacity (GGO) appearance with air bronchograms and pleural retraction on CT imaging. Haematoxylin–eosin staining (**c**) shows the lepidic predominant histological type of adenocarcinoma. The results of the amplification-refractory mutation system (ARMS) method (**d**) identified an L858R mutation in exon 21 of the EGFR gene in the tumour.

**Figure 3:**
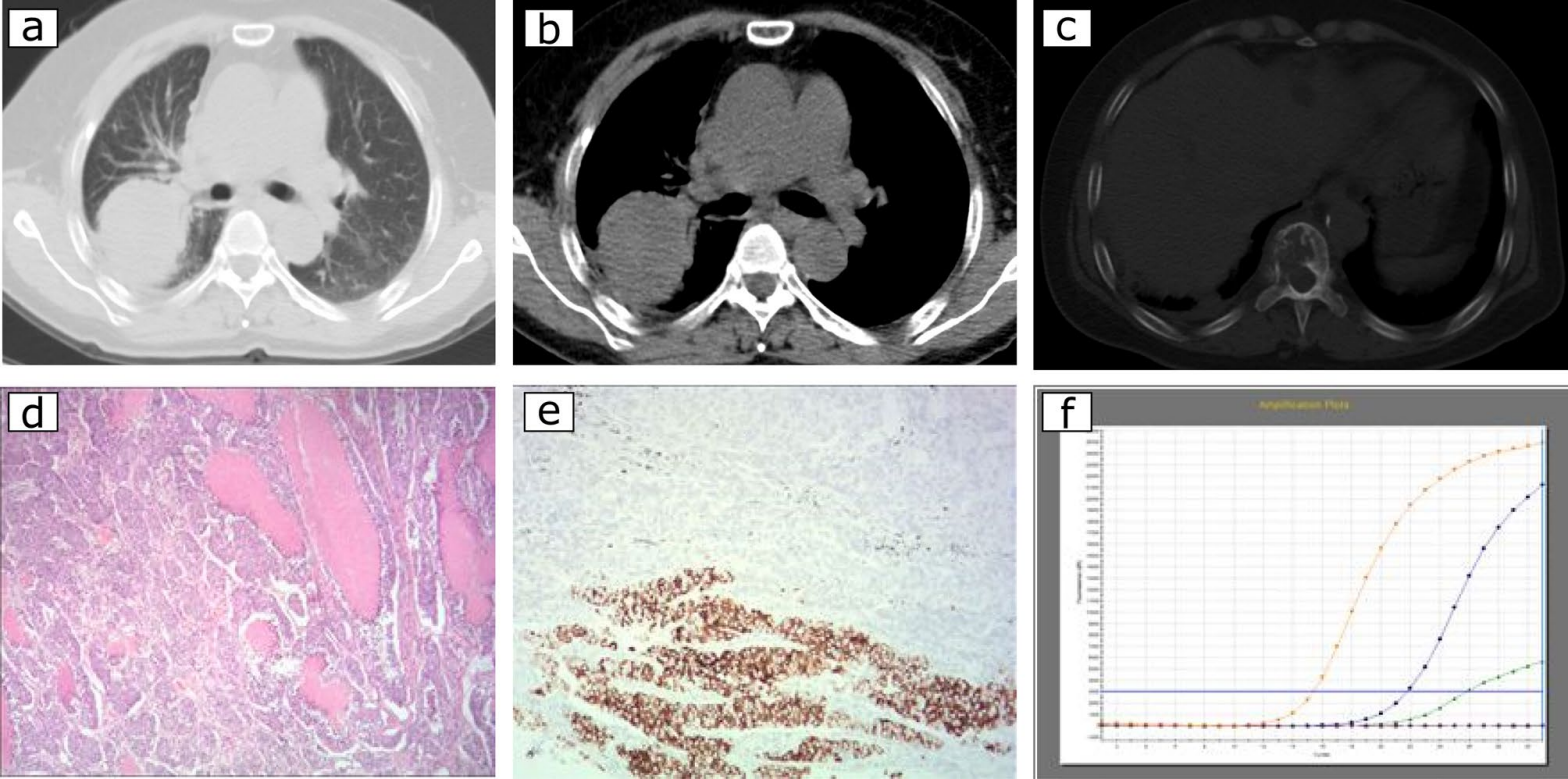
A 61-year-old female with primary lung adenocarcinomas in the right lower lobe (**a**,**b**) who presented as a lobulated solid tumour with concomitant EGFR mutation and ALK rearrangement positive. Th12 vertebral bone metastasis can be seen in the bone window (**c**). Haematoxylin–eosin staining (**d**) shows a solid predominant histological type of adenocarcinoma. The results of immunohistochemistry (IHC) the tumour was positive (**e**); the ARMS method (**f**) identified EGFR mutations in exons 19 and 20 in the tumour.

In regard to radiologic features, tumours with EGFR mutations tended to be smaller than EGFR-wild-type tumours (*p* < 0.001) (Fig. 1b), and the optimal cut-off value of the tumour maximum diameter was 33 mm (Fig. 1d). EGFR-mutated tumours also tended to have peripheral lesions (*p* = 0.035) and GGOs (*p* < 0.001), especially mixed GGOs (mGGOs) (*p* = 0.045), well-defined margins (*p* = 0.022), air bronchograms (*p* < 0.001), pleural retraction (*p* < 0.001) and no lymphadenopathy (*p* = 0.001) (Fig. 2). No other CT signs were associated with EGFR mutation status (Table 4). ALK positivity was associated with solid tumours (*p* = 0.009) (Fig. 3) and the absence of air bronchograms (*p* < 0.001) and with metastasis (*p* < 0.001) (Fig. 3). No other CT signs were associated with ALK rearrangement status (Table 4).

**Table 4:** Association between CT features and EGFR and ALK status in adenocarcinoma.

Variable	EGFR + N (%)	EGFR − N (%)	Total	*P*	ALK + N(%)	ALK − N(%)	Total	*P*
Tumour size (mm)^&^	26 ± 13	30 ± 17	28 ± 15	< 0.001*	26 ± 13	28 ± 15	28 ± 15	0.304
Type				0.035*				0.762
Central	18 (4)	24 (7)	42 (5)		2 (4)	40 (5)	42 (5)	
Peripheral	471 (96)	314 (93)	785 (95)		55 (96)	730 (95)	785 (95)	
Location				0.036*				0.006*
Left upper lobe	102 (21)	72 (21)	174 (21)		22 (39)	152 (20)	174 (21)	
Left lower lobe	81 (17)	39 (12)	120 (15)		7 (12)	113 (15)	120 (15)	
Right upper lobe	163 (33)	129 (38)	292 (35)		13 (22)	279 (36)	292 (35)	
Right middle lobe	30 (6)	27 (8)	57 (7)		1 (2)	58 (8)	57 (7)	
Right lower lobe	113 (23)	71 (21)	184 (22)		16 (28)	168 (22)	184 (22)	
Texture				< 0.001*^¥^				0.009*^¥^
Solid	357 (73)	302 (89)	659 (80)		53 (93)	606 (79)	659 (80)	
GGO	132 (27)	36 (11)	168 (20)	< 0.001*^,^!	4 (7)	164 (21)	168 (20)	0.034*^,^ !
pGGO	20 (4)	9 (3)	29 (4)	0.106^	0 (0)	29 (2)	29 (4)	0.048*^,^^
mGGO	112 (23)	27 (8)	139 (16)	< 0.001*^,#^	4 (7)	132 (19)	139 (16)	0.024*^, #^
Shape				0.439				1.000
Lobulated	408 (83)	289 (86)	697 (84)		48 (84)	649 (84)	697 (84)	
Other	81 (17)	49 (14)	130 (16)		9 (16)	121 (16)	130 (16)	
Margin				0.524				1.000
Smooth	255 (52)	168 (50)	423 (51)		29 (51)	394 (51)	423 (51)	
Spiculate	234 (48)	170 (50)	404 (49)		28 (49)	376 (49)	404 (49)	
Margin definition				0.022*				0.285
Well-defined	150 (31)	79 (23)	229 (28)		12 (21)	217 (28)	229 (28)	
Poor-defined	339 (69)	259 (77)	598 (72)		45 (79)	553 (72)	598 (72)	
Air bronchogram	208 (43)	62 (18)	270 (33)	< 0.001*	5 (9)	265 (34)	270 (33)	< 0.001*
Heterogeneity	311 (64)	194 (57)	505 (61)	0.082	32 (56)	473 (61)	505 (61)	0.483
Pleural retraction	348 (71)	183 (54)	531 (64)	< 0.001*	33 (58)	498 (65)	531 (64)	0.318
Pleural effusion	21 (4)	23 (7)	44 (5)	0.114	5 (9)	39 (5)	44 (5)	0.229
Pleural thickening	34 (7)	35 (10)	69 (8)	0.325	2 (4)	66 (9)	69 (8)	0.171
Calcification	19 (4)	20 (6)	39 (5)	0.175	2 (4)	37 (5)	39 (5)	1.000
Cavitation	25 (5)	27 (8)	52 (6)	0.094	4 (7)	48 (6)	52 (6)	0.814
Bubble-like lucency	71 (15)	41 (12)	112 (14)	0.353	6 (11)	106 (14)	112 (14)	0.687
Necrosis	88 (18)	75 (22)	163 (20)	0.136	9 (16)	154 (20)	163 (20)	0.495
Vascular convergence	103 (21)	75 (22)	178 (22)	0.201	10 (18)	168 (22)	178 (22)	0.446
Peripheral fibrosis	151 (31)	96 (28)	247 (30)	0.444	23 (40)	224 (29)	247 (30)	0.073
Peripheral emphysema	78 (16)	71 (21)	149 (18)	0.063	10 (18)	139 (22)	149 (18)	0.923
Enhancement				0.850				0.670
Mild	95 (46)	72 (47)	167 (46)		14 (58)	153 (46)	167 (46)	
Moderate	66 (32)	52 (34)	118 (33)		6 (25)	110 (33)	118 (33)	
Marked	45 (22)	30 (19)	75 (21)		4 (17)	71 (21)	75 (21)	
Lymphadenopathy	85 (17)	92 (27)	177 (21)	0.001*	14 (25)	163 (21)	177 (21)	0.508
Distant metastasis	175 (36)	131 (39)	306 (37)	0.384	37 (75)	229 (30)	306 (37)	< 0.001*

* *P* values < 0.05 were based on comparisons between the two groups; ^&^ The maximum diameter of the lesion (in centimetres) evaluated on multiplanar reconstructions (MPRs) with a soft tissue window; ¥Comparison between solid and GGO.

^Comparison between solid and pGGO.

^#^ Comparison between solid and mGGO.

!Comparison between pGGO and mGGO.

*EGFR* epidermal growth factor receptor; *ALK* anaplastic large-cell lymphoma kinase; *EGFR*+ EGFR mutation; *EGFR*−, EGFR wild-type mutation; *ALK*+ ALK positive; ALK− ALK negative.

*GGO* ground-glass opacity; *pGGO* pure ground-glass opacity; *mGGO* mixed ground-glass opacity.

### Differences in CT features between EGFR exon 21 and -19 mutations

The exon 21 mutation rate in GGOs was significantly higher than the exon 19 mutation rate (34% vs 24%, *p* = 0.029). However, no differences in sex, smoking history, predominant subtype, type, tumour maximum diameter, air bronchogram, margin, or lymphadenopathy were found between patients with EGFR exon 19 and exon 21 mutations (Table 5).

**Table 5:** Differences in clinical and CT features between exon 21 and 19 EGFR-mutated adenocarcinoma.

EGFR mutation	Exon 21 (N = 242) n (%)	Exon 19 (N = 215) n (%)	*P* value
Gender			0.960
Male	94 (39)	84 (39)	
Female	148 (61)	131 (61)	
Smoking history	61 (25)	45 (21)	0.280
Predominant subtypes			0.070
Lepidic predominant^$^	29 (12)	15 (7)	
Others subtypes^@^	213 (88)	200 (93)	
Maximum diameter (mm)	25.8	26.2	0.781
Type			1.000
Central	10 (4)	8 (4)	
Peripheral	232 (96)	207 (96)	
Texture			0.029*
Solid	161 (67)	163 (76)	
GGO	81 (34)	52 (24)	
Air bronchogram	106 (44)	89 (42)	0.553
Margin			1.000
Well-defined	168 (69)	150 (70)	
Poor-defined	74 (31)	65 (30)	
Lymphadenopathy	65 (27)	41 (19)	0.052

* *P* values < 0.05 were based on comparisons between the two groups;

GGO, ground glass opacity.

^$^ Lepidic predominant includes adenocarcinoma in situ, minimally invasive adenocarcinoma, and lepidic predominant invasive adenocarcinoma; @ other subtypes include acinar, papillary, micropapillary, and solid predominant adenocarcinoma, as well as variants of invasive mucinous adenocarcinoma.

### Multivariable and ROC curve analyses of prognostic factors for EGFR mutations

**Figure 4:**
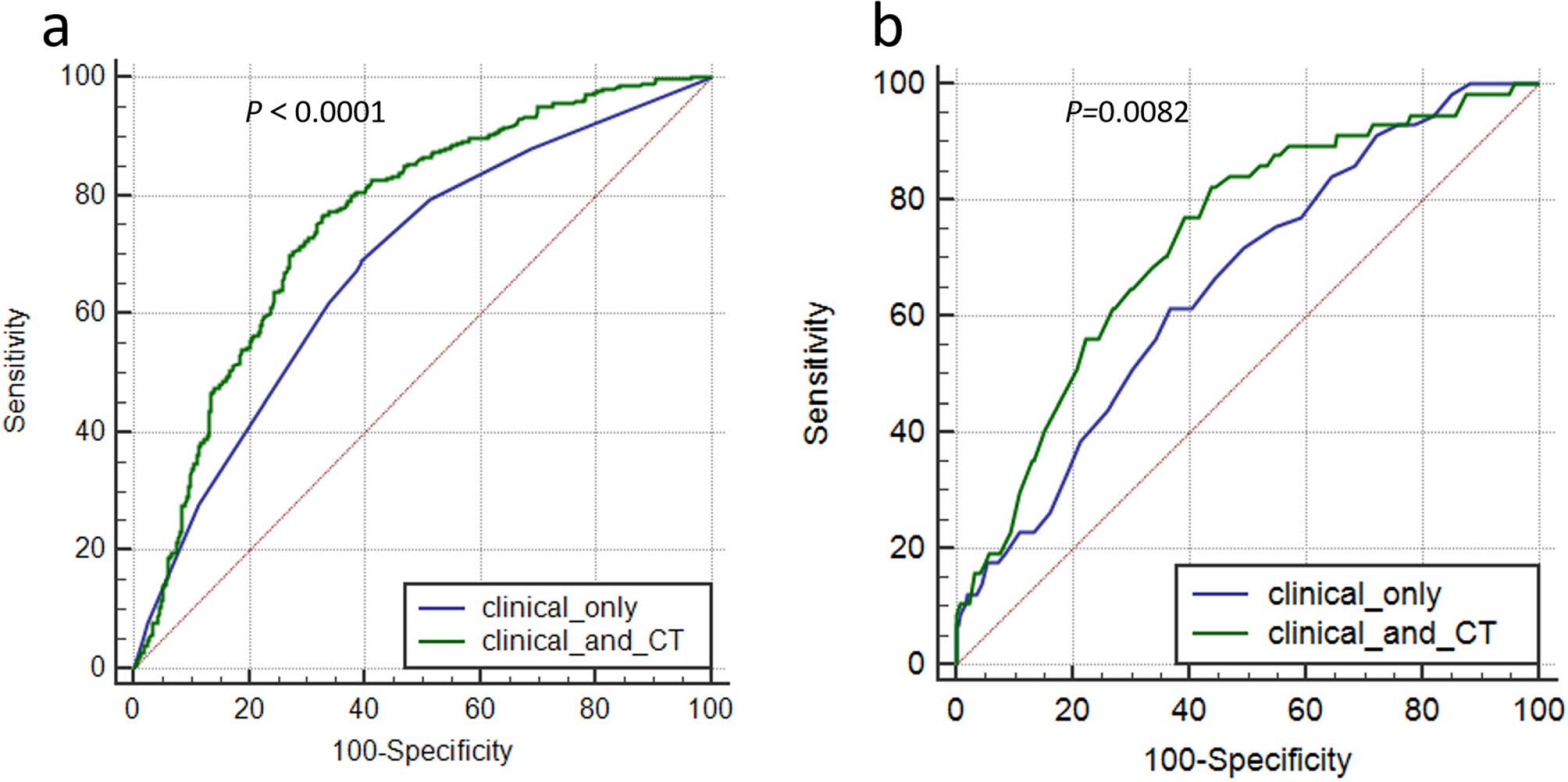
(**a**) Receiver operating characteristic curves for the prediction of EGFR mutation status using clinical variables alone and combined with CT features in patients with lung adenocarcinoma. (**b**) Receiver operating characteristic curves for the prediction of ALK-positive status using clinical variables alone and combined with CT features in patients with lung adenocarcinoma.

In the model including both clinical variables and CT features, regression showed that the most significant independent prognostic factors of EGFR + were female sex (OR = 1.713, 95% CI:1.117, 2.628), non-smoking status (OR = 0.557, 95% CI: 0.357,0.868), GGO (OR = 3.035, 95% CI: 1.841, 5.004), air bronchograms (OR = 1.912, 95% CI: 1.336, 2.737) and pleural retraction (OR = 2.183, 95% CI:1.557, 3.061) (Table 6). ROC curve analysis yielded area under the curve (AUC) values of 0.682 and 0.758 for clinical only or combined CT features, respectively, for the prediction of EGFR mutation, and a significant difference was found between them (*p* < 0.0001). (Fig. 4a).

**Table 6:** Logistic regression analyses of various predictive factors for EGFR mutation in adenocarcinoma.

Variables	B	Wald X2	*P* value	OR	95% CI
Gender	0.538	6.076	0.014	1.713	1.117–2.628
Smoking status	− 0.586	6.690	0.010	0.557	0.357–0.868
Lepidic predominant$	0.493	1.495	0.221	1.637	0.743–3.609
lymphatic metastasis	− 0.189	0.993	0.319	1.208	0.833–1.751
maximum diameter	0.000	0.003	0.957	1.000	0.989–1.012
Type	− 0.188	0.271	0.603	0.829	0.408–1.683
Texture	1.110	18.935	0.000	3.035	1.841–5.004
Air bronchograms	0.648	12.566	0.000	1.912	1.336–2.737
Margin	0.062	0.104	0.747	1.063	0.732–1.545
Pleural retraction	0.781	20.502	0.000	2.183	1.557–3.061
lymphadenopathy	− 0.307	2.005	0.157	0.736	0.481–1.125

*OR* odds ratio; *CI* confidence interval.

^$^Lepidic predominant includes adenocarcinoma in situ, minimally invasive adenocarcinoma, and lepidic predominant invasive adenocarcinoma.

### Multivariable and ROC curve analyses of prognostic factors for ALK mutations

In logistic regression analysis, age (OR = 0.940, 95% CI: 0.910, 0.972), tumours with the solid-predominant subtype or mucinous lung adenocarcinoma (OR = 7.994, 95% CI: 4.183, 15.278), solid tumours (OR = 0.292, 95% CI: 0.097, 0.878) and lesions without air bronchograms (OR = 0.307, 95% CI: 0.123, 0.767) were significantly associated with ALK positivity (Table 7). ROC curve analysis showed that the use of clinical variables combined with CT features (AUC = 0.739) was superior to the use of clinical variables alone (AUC = 0.657) for the prediction of ALK positivity, and a significant difference was found between them (*p* = 0.0082) (Fig. 4b).

**Table 7:** Logistic regression analyses of various predictive factors for ALK-positive status in adenocarcinoma.

Variables	B	Wald X^2^	*P* value	OR	95% CI
Age	− 0.062	13.391	0.000	0.940	0.910–0.972
Solid predominant or Mucinous	2.079	39.570	0.000	7.994	4.183–15.278
Texture	− 1.230	4.806	0.028	0.292	0.097–0.878
Air bronchograms	− 1.180	6.389	0.011	0.307	0.123–0.767

OR, odds ratio; CI, confidence interval.

### Correlation analysis of histopathology subtype with lesion texture on CT

Tumours displaying GGOs on CT correlated positively with the lepidic-predominant subtype (*β* = 0.325, *p* < 0.001). A positive correlation was also found between lesions with a solid appearance on CT and the solid-predominant subtype or mucinous adenocarcinoma (*β* = 0.363, *p* < 0.001).

## Discussion

The EGFR mutation rate has been reported to be 27–56% in Asian patients^13,21,22^. Patients had a similar EGFR mutation rate in our study (59.1%), mainly composed of exons 19 and 21 (93.5%). Previous studies have reported that females and nonsmokers have an increased risk of EGFR mutations^17,23^, which was confirmed in our series. A recent study found that EGFR mutations more commonly occurred in early-stage NSCLC patients than in advanced-stage patients^24^. The present study showed that the mutation rate of EGFR (55.6%) in lung adenocarcinoma of TNM stage I-II was similar to that in stage III-IV (56.5%). Such a discrepancy in results may be due to individual differences in the samples included between studies. Moreover, in our study, EGFR mutations were associated with a small tumour size and lymph node metastasis proven by surgery, suggesting a low TNM stage in those cases. In addition, our study demonstrated that EGFR mutations were significantly more common in lepidic predominant adenocarcinomas^13,14^. This result is supported by gene expression profiling microarray studies in which high EGFR mutation frequencies were observed in terminal respiratory unit adenocarcinoma^13,25^.

Several studies have explored the correlation between EGFR gene mutations and GGOs on CT^11–15^. For example, Glynn et al. and Sugano et al.^11,12^ found no significant association between GGOs and EGFR mutations, whereas Liu et al.^13^ reported that a GGO appearance and 15 other CT features were significantly associated with EGFR mutations. In the present study, three main results regarding correlations between GGO and EGFR mutations were identified. First, EGFR mutations were more frequently associated with GGOs by CT, consistent with most previous studies^10,13,17^. This finding may be due to the inverse relationship between the replication of EGFR and the percentage of GGOs on CT^26,27^. Moreover, this result was supported by the pathological-imaging correlation in this study: tumours displaying GGOs on CT correlated positively with the lepidic-predominant subtype. Second, our series showed that mixed GGO (mGGO) lesions are more susceptible to EGFR mutations than pure GGO (pGGO) or solid lesions. Hsu et al.^15^ stated that EGFR mutations are more common in invasive solid patterns and significantly less common in pGGO patterns in stage I lung adenocarcinoma. Possible explanations for the above findings may be that EGFR mutations promote the conversion of pGGO to mGGO and indicate more aggressive behaviour. Third, GGOs were significantly more highly related to the exon 21 mutation rate than to the exon 19 mutation rate, in line with Lee et al.^14^. The results indicated that GGO is not only a factor for EGFR mutation but also an incidence factor for EGFR exon 21 mutation. Because TKIs show different targeted effects in cases of EGFR exon 21 and -19 mutations^20^, analysis of clinical or imaging variables between these cases can provide a comprehensive baseline for selecting targeted treatment.

In the present study, patients with EGFR mutations frequently presented with smaller tumours (tumour maximum diameter ≤ 33 mm) and peripheral lesions, although Liu et al.^13^ found that EGFR mutations are more common in peripheral lung adenocarcinomas with diameters < 3 cm. This difference may be attributed to the different methods used between the studies, as the cut-off was self-defined in the previous study^13^. In our study, ROC curve analysis was used to determine the cut-off. In addition, our study found associations between EGFR mutations, air bronchogram and pleural retraction, which agrees with Zhou et al.^17^ and Stefania et al.^16^ but contradicts Mizue et al.^28^. These controversial results may be the result of different ethnicities, grouping methods and sample sizes. Air bronchograms reflect tumour invasion or expansion without destroying the intratumoural bronchus, suggesting reduced aggressiveness. Pleural retraction is a common sign of visceral pleural invasion, which is one of the most important prognostic factors after the surgical resection of NSCLC^29^. Nevertheless, in the present study, pleural invasion (proven by surgery) did not differ between wild-type and mutant EGFR. The reason for this difference may be that pleural retraction does not always mean pleural invasion pathologically. Besides, our study showed that EGFR mutation was associated with less lymphadenopathy and less lymph node metastasis pathologically, supporting a previous study^13^, suggesting a lower invasiveness of tumours with EGFR mutations.

ALK rearrangement has been identified in 0.4 to 13.5% of unselected NSCLC patients^30,31^, consistent with the present study (7.0%). Previous studies have reported that patients with ALK positivity tend to be younger and are more often never smokers than patients with ALK-negative tumours^32^. We found that a younger age (≤ 56 years old) was associated with ALK positivity. However, no significant difference in smoking status was found. A recent report by Li et al.^33^ conducted on a relatively large sample demonstrated that ALK rearrangements are more commonly observed in the solid predominant subtype of adenocarcinoma. A high frequency of solid growth or mucus patterns in ALK-positive tumours was observed in the present study, consistent with the above report. Previous studies have demonstrated that ALK positivity is more common in stage IV disease (ranging from 9.7–28.0%). In contrast, ALK positivity showed no significant difference between the stage I-II and III-IV groups in our study. We indeed found that ALK positivity was associated with metastasis detected by CT, suggesting a higher TNM staging.

A limited number of reports have focused on the association between ALK positivity and CT features^16–19^ because of the low rate of ALK positivity in lung adenocarcinoma. According to Zhou et al. and Chang et al.^17,19^, a solid pattern is the main characteristic of ALK-positive tumours. Zhou et al.^17^ also found that a pure GGO appearance was significantly less common in ALK-positive cases than in EGFR mutation cases. Similarly, in our series, ALK positivity was associated with solid nodules or masses without GGOs on CT. This imaging finding was consistent with the correlation between solid lesions on CT and solid-predominant subtypes or mucinous adenocarcinoma in the present study, whereas ALK positivity commonly occurred in tumours of solid-predominant subtypes or mucus patterns. Therefore, the correlation between CT signs and gene mutations may be due to different histological growth characteristics.

Previous studies have reported that ALK-positive lung adenocarcinoma frequently occurs with extensive lymph node metastasis^18,19^, pleural retraction^17^, pleural effusion^16^, and distant metastasis^34^. Among them, distant metastasis in ALK-positive patients was supported by our study, although we did not find significant correlations between ALK positivity and other CT features, which may be caused by the small sample size in the current studies. In addition, the present study found that ALK-positive tumours lack air bronchograms. As mentioned above, the sign of air bronchogram represents reduced tumour invasiveness. When taken together, we may reasonably consider that ALK-positive lung adenocarcinoma is associated with high invasion. The lack of GGO manifestations in our study also supports this biological behaviour.

Our study has some limitations. First, this study was conducted at a single institution, and the patients in our study were all Chinese and thus had a genetic alteration pattern distinct from that of other races, which may impede the application of our results to other ethnicities. To improve the generalization ability and optimization of the model, multidisciplinary and prospective research is needed. Second, due to the limitation of the retrospective analysis method, our study is just a preliminary research study on CT features for predicting gene mutations. However, this report serves as a basis for comprehensive and prospective investigations analysing these patient populations. Third, although we strictly used double-blind methods to record CT signs, EGFR and ALK status, selection bias was inevitable. Finally, the present study analysed only adenocarcinoma and did not include other histologic subtypes, which could explain the results. However, this is understandable, as the majority of EGFR mutations and ALK positivity are found in adenocarcinomas, with an extremely low mutation rate in squamous cell carcinoma (< 5%)^35^. Finally, CT findings of distance metastases were not pathologically confirmed. Thus, we did not include distance metastases in the multivariate logistics analysis to predict ALK positivity.

In conclusion, combining clinical variables and CT features was more effective in predicting EGFR and ALK than using clinical variables alone. In addition, GGO is not only a factor for EGFR mutation but also an incidence factor for EGFR exon 21 mutation. Therefore, the use of CT features for patients can allow analyses of tumours and more accurately predict patient populations who will benefit from EGFR-TKIs or ALK crizotinib treatment.

## Materials and methods

### Patients and inclusion criteria

A total of 1,459 patients evaluated by the multidisciplinary thoracic oncology group between January 2010 and February 2017 at the Union Hospital of Tongji Medical College were retrospectively screened. Initially, 1,186 patients were included according to the inclusion criteria: (1) lung adenocarcinoma confirmed by surgical resection; (2) available pathology reports (including predominant pathological subtypes, lymph node metastasis and pleural invasion, etc.); (3) available results for both EGFR mutations and ALK status; and (4) available clinical data. In total, 359 patients were excluded because of the following three exclusion criteria: (1) thin-section CT was not available (n = 251); (2) heavy CT image artefacts (n = 65); and (3) received preoperative treatment with chemotherapy or radiation therapy before surgery (n = 43). Ultimately, 827 patients were included. The patient’s clinical characteristics, including age, sex, smoking history, histopathology, nodal involvement, and tumour stage, among others, were recorded. In accordance with Lv et al.^34^, nonsmoking was defined as lifetime exposure to fewer than 100 cigarettes, and the remaining patients were categorized as ever-smokers. TNM staging was based on the IASLC 8th TNM Lung Cancer Staging System^36^. This retrospective study was approved by the Institutional Review Board of Union Hospital of Tongji Medical College. All subjects enrolled signed a written consent form after being informed of the details of the research. This study was conducted in compliance with the Declaration of Helsinki.

### EGFR mutation analysis

EGFR mutations were analysed according to the principle of the amplified drug resistance mutation system (ARMS). Primary tumours or lymph nodes were simply excised, aspirated, or biopsied, followed by 10% neutral buffered formalin fixation and paraffin embedding. DNA was extracted from formalin-fixed paraffin-embedded (FFPE) tissue sections, and the Qiagen FFPE Tissue Kit (Netherlands Roots NV) was used according to the manufacturer's instructions. PCR was carried out using the Mx3000PTM system (Stratagene, La Jolla, USA) with an EGFR 29 Mutations Detection Kit (Amoy Diagnostics, Xiamen, People’s Republic of China), and the results were interpreted according to the manufacturer’s instructions. Molecular analysis of EGFR mutations was defined as mutations in EGFR exons 18, 19, 21, or 20; other types of EGFR mutations were defined as wild-type EGFR^5,37^.

### VENTANA ALK immunohistochemical (IHC) assay

VENTANA is a fully automated IHC detection method based on the monoclonal antibody D5F3. The VENTANA IHC assay has been approved by the US FDA and the China FDA for the identification of patients with NSCLC who are eligible for treatment with ALK TKIs. FFPE tissue sections with a thickness of 4 μm were cut according to the manufacturer's instructions and a scoring algorithm. The result was dichotomous, whereby the presence of any percentage of positive tumour cells with strong granular cytoplasmic staining was regarded as ALK positivity and all other observations were regarded as ALK negativity.

### CT image acquisition

CT was performed at our institution using a multislice spiral CT system (SOMATOM Definition AS + , Siemens Healthineers, Germany)^38^. The scan ranged from the level of the chest inlet to the inferior level of the costophrenic angle. The CT parameters were as follows: detector collimation width, 64 × 0.6 mm and 128 × 0.6 mm; tube voltage, 120 kV. The tube current was regulated by an automatic exposure control system (CARE Dose 4D). Images were reconstructed with a slice thickness of 1.5 mm and an interval of 1.5 mm. The reconstructed image is transmitted to the workstation and picture archiving and communication systems (PACS) for multiplanar reconstruction (MPR) postprocessing. Nonionic iodine contrast agents (60–80 ml iohexol 350 mg/mL, Beilu Pharmaceutical Co., Ltd.; Beijing, China) at a dose of 3 Ml/s were intravenously injected into 360 patients.

Two radiologists with different degrees of experience in interpreting chest CT images independently performed all qualitative image analyses^38^. One of them was a senior radiologist with 10 years of chest imaging (H.S.); the other is a fellow with 4 years of experience in CT image interpretation (J.G.). Both analysed the Digital Imaging and Communications in Medicine (DICOM) images from the CT studies without access to clinical and histologic findings but were aware of the presence and sites of tumours. They assessed CT features using both axial CT images and MPR images. After separate evaluations were performed, differences were resolved by consensus. For each CT scan, the data shown in Table 1 were recorded.

### Statistical analysis

The analyses were performed using SPSS Statistics (SPSS, version 21, IBM, Chicago, IL, USA) and MedCalc 16.2.0 (MedCalc Software, Mariakerke, Belgium)^38^. Distribution normality was assessed using the Kolmogorov–Smirnov test. Normally and nonnormally distributed data and categorical variables are expressed as the mean ± standard deviation, median (interquartile range) and frequency (percentage), respectively. The independent-sample Student’s *t* test was applied to compare two groups of normally distributed variables, and one-way ANOVA and the chi-square test were used to compare categorical variables. Multivariate linear regression analyses (binary logistic regression) were performed to identify independent factors predictive of EGFR or ALK mutation status. The final model was selected by using the enter elimination method, with a cut-off *P* value of 0.05. A *P* value < 0.05 (two-tailed) was considered to be statistically significant. Receiver operating characteristic (ROC) curves were constructed for the ability of combined independent factors to predict EGFR mutations or ALK positivity. Comparison of the ROC curves for clinical characteristics alone and clinical characteristics combined with CT signs was performed by the nonparametric approach of DeLong et al. Patient age and the tumour maximum diameter were applied to examine the diagnostic performance of ALK positivity and EGFR mutation by ROC curve analysis. The sensitivity, specificity and optimal cut-off value were calculated. The repeatability test of the maximum tumour diameter was evaluated by intraclass correlation coefficient (ICC) analysis and the 95% CI. For other CT signs, interobserver agreement was assessed by the *k* coefficient. Pearson’s correlation was used to analyse the relationship between histopathology subtype and lesion texture. A *P* value < 0.05 (two-tailed) was considered to be statistically significant.

## Ethics declarations

This study was approved by the ethics committee of Tongji Medical College of Huazhong University of Science and Technology. All subjects provided written informed consent.

## Data Availability

The datasets used and/or analysed during the current study are available from the corresponding author on reasonable request.

## Abbreviations

CT: Computed tomography
EGFR: Epidermal growth factor receptor
ALK: Anaplastic large-cell lymphoma kinase
NSCLC: Nonsmall cell lung carcinoma
TKIs: Tyrosine kinase inhibitors
GGOs: Ground-glass opacities
pGGO: Pure GGO
mGGO: Mixed GGO
PFS: Progression-free survival
TNM: Tumour node metastasis
ROC: Receiver operating characteristic
AUC: Area under the curve
IASLC: International Association for the Study of Lung Cancer
ARMS: Amplified drug resistance mutation system
IHC: Immunohistochemistry
FFPE: Formalin-fixed paraffin-embedded
FISH: Fluorescence in situ hybridization
